# Metabolic Profiling in Maturity-Onset Diabetes of the Young (MODY) and Young Onset Type 2 Diabetes Fails to Detect Robust Urinary Biomarkers

**DOI:** 10.1371/journal.pone.0040962

**Published:** 2012-07-30

**Authors:** Anna L. Gloyn, Johan H. Faber, Daniel Malmodin, Gaya Thanabalasingham, Francis Lam, Per Magne Ueland, Mark I. McCarthy, Katharine R. Owen, Dorrit Baunsgaard

**Affiliations:** 1 Oxford Centre for Diabetes, Endocrinology & Metabolism, University of Oxford, Oxford, United Kingdom; 2 Oxford NIHR Biomedical Research Centre, Churchill Hospital, Oxford, United Kingdom; 3 Novo Nordisk A/S, Novo Nordisk, Måløv, Denmark; 4 Department of Clinical Biochemistry, John Radcliffe Hospital, Oxford, United Kingdom; 5 Section for Pharmacology, University of Bergen, Bergen, Norway; 6 Wellcome Trust Centre for Human Genetics, University of Oxford, Oxford, United Kingdom; Hirosaki University Graduate School of Medicine, Japan

## Abstract

It is important to identify patients with Maturity-onset diabetes of the young (MODY) as a molecular diagnosis determines both treatment and prognosis. Genetic testing is currently expensive and many patients are therefore not assessed and are misclassified as having either type 1 or type 2 diabetes. Biomarkers could facilitate the prioritisation of patients for genetic testing. We hypothesised that patients with different underlying genetic aetiologies for their diabetes could have distinct metabolic profiles which may uncover novel biomarkers. The aim of this study was to perform metabolic profiling in urine from patients with MODY due to mutations in the genes encoding glucokinase (GCK) or hepatocyte nuclear factor 1 alpha (HNF1A), type 2 diabetes (T2D) and normoglycaemic control subjects. Urinary metabolic profiling by Nuclear Magnetic Resonance (NMR) and ultra performance liquid chromatography hyphenated to Q-TOF mass spectrometry (UPLC-MS) was performed in a Discovery set of subjects with HNF1A-MODY (n = 14), GCK-MODY (n = 17), T2D (n = 14) and normoglycaemic controls (n = 34). Data were used to build a valid partial least squares discriminate analysis (PLS-DA) model where HNF1A-MODY subjects could be separated from the other diabetes subtypes. No single metabolite contributed significantly to the separation of the patient groups. However, betaine, valine, glycine and glucose were elevated in the urine of HNF1A-MODY subjects compared to the other subgroups. Direct measurements of urinary amino acids and betaine in an extended dataset did not support differences between patients groups. Elevated urinary glucose in HNF1A-MODY is consistent with the previously reported low renal threshold for glucose in this genetic subtype. In conclusion, we report the first metabolic profiling study in monogenic diabetes and show that, despite the distinct biochemical pathways affected, there are unlikely to be robust urinary biomarkers which distinguish monogenic subtypes from T2D. Our results have implications for studies investigating metabolic profiles in complex traits including T2D.

## Introduction

Monogenic disorders of beta-cell function (maturity-onset diabetes of the young; MODY) have a minimum estimated prevalence of approximately 100 cases per million population [1], [2]. There are significant clinical implications for both patients and their relatives associated with finding a genetic aetiology underlying their diabetes and it is therefore important to identify these individuals [3], [4]. Diagnostic molecular testing is widely available, however inequality in referral rates mean that a significant proportion (>80%) of cases go undiagnosed and are therefore misclassified as either type 1 (T1D) or type 2 diabetes (T2D) [2]. The principle barriers to genetic testing include the cost of DNA sequencing which prohibits indiscriminate use. Current recommendations for genetic testing [5] are based on non-specific clinical criteria such as age of onset of diabetes, family history and an atypical clinical presentation for either T1D or T2D which have a high specificity but low sensitivity [6]. Robust cost-effective biomarkers which could be used as an adjunct to current tests to prioritise patients for molecular genetic testing could help address this problem.

The most common subtypes of MODY in all populations studied are the result of mutations in the genes encoding the transcription factor Hepatocyte nuclear factor 1 alpha (HNF1A) and the key glycolytic enzyme glucokinase (GCK) [5]. HNF1A is expressed in a number of tissues including pancreas and liver where it plays an important role in the regulation of a large number of genes [7]. It is the role of HNF1A in liver which provides the greatest potential for urinary and/or plasma biomarkers, which could aid the differentiation from other forms of diabetes.

There have been several studies evaluating candidate biomarkers which have emerged from rodent models and human genetics but until recently none have demonstrated sufficient specificity and sensitivity and/or withstood replication in other populations [8], [9], [10], [11], [12], [13], [14], [15], [16]. The most promising biomarker to date is highly sensitive C-reactive protein (hsCRP) [6], [11], [17]. Although these data are promising, one significant limitation of CRP as a biomarker is that it is an acute phase protein and levels are raised during infection [11]. Metabolomics aims/endeavours to provide a global assessment of metabolites in a given sample and has recently been used to investigate *Hnf1a1*-dependent pathways in urine samples from *Hnf1a^−/−^* null and wild-type mice [18]. More recently, metabolic profiling has been proposed as a platform for identifying metabolites which serve as accurate indicators of diabetes pathogenesis and progression [19], [20]. Given the different genetic aetiologies and distinct metabolic pathways affected, we hypothesised that subjects with HNF1A-MODY could have a different pattern of urinary metabolic profile compared to subjects with GCK-MODY and T2D. The first aim of this study was to determine the metabolic profiles of subjects with monogenic diabetes and identify metabolites which distinguish the different genetic subtypes (Discovery Study) and second to evaluate potential biomarkers in an extended group of subjects.

## Results

### Metabolic profiling of diabetic subtypes by Ultra Performance Liquid Chromatography Mass Spectrometry (UPLC-MS)

We compared the metabolic urine profile of samples from normoglycaemic control and T2D subjects with samples from HNF1A-MODY and GCK-MODY subjects. The clinical characteristics of the subjects studied are given in table 1. Urine samples were analysed using two different platforms; liquid chromatography mass spectrometry (LC-MS) and ^1^H nuclear magnetic resonance spectroscopy (NMR).

**Table 1:** Clinical Characteristics of Subjects in the Discovery and extended datasets.

	Discovery dataset				Extended dataset	
	HNF1A-MODY n = 25	GCK-MODY n = 24	T2D n = 14	Non-diabetic n = 34	HNF1A-MODY n = 39	T2D n = 158
**Gender M:F %**	36:64	42:58	50:50	53:47	33:67	57:43
**Age at sampling years**	38.3(26.9)	25.1(11.4)	52.7(12.1)	49(8.5)	44.2(24.0)	52.4(18.2)
**Duration of diabetes** **years**	11.9(24.9) 4 subjects non-diabetic	Hyperglycaemia from birth	3.8(3.5)	N/A	21.5(29.1) 6 subjects non-diabetic	14.8(16.9)
**Treatment** **Diet:OHA:Insulin %**	14:43:38	79:21:0	100:0:0	N/A	10:43:47	3:29:68
**Fasting glucose mmolL^−1^**	8.3(3.4)	7.1(1.1)	6.3(2.4)	5.1(0.4)	6.9 (4.1)	8.5(3.8)
**HbA1c %**	7 (1.4)	6.5(0.7)	6.1(0.7)	Not available	7.1(1.4)	7.6(2.0)
**BMI kgm^−2^**	24.3(4.1)	25.1(11.4)	31.4(8.5)	24.7(3.4)	24.2(4.7)	33.1(9.4)

Data is shown as median (IQR) or %.

The LC-MS based method acquired approximately 3000 MS peaks of positive ions (ESI^+^). Representative positive base peak intensity (TBPI) chromatograms of the controls and the three diabetic subgroups are shown in Figure S1. Maximum separation between the four defined classes in the data (controls, T2D, HNF1A-MODY and GCK-MODY) was assessed using partial least squares regression discriminant analysis (PLS-DA) models. The models were built from 1752 peaks of normalized MS data after eliminating the background and non-biologically relevant peaks. A four-class model of controls versus the three diabetic subgroups as separate classes showed a reasonable separation between the controls and the three diabetic groups, but the model was not valid in a permutation test (Table S1). A two-class model of the controls versus all diabetic subjects as one group proved to be valid with a Q^2^ above 0.5 (Table S1 and Figure S2). Three non-diabetic *HNF1A* mutation carriers and a mutation carrier with impaired glucose tolerance (IGT) were included with the HNF1A subjects selected for the Discovery study. These four subjects were first predicted into the two-class model, but since they all grouped with the diabetic subjects in the model, these four samples were included in HNF1A-MODY group in all subsequent analyses (red and blue boxes in Figure S2).

**Figure 1:**
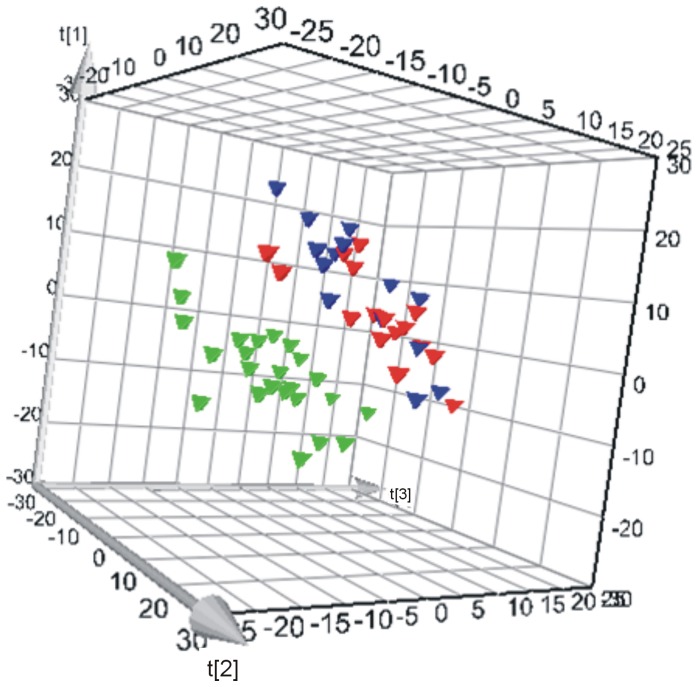
3D score plot of a two-class PLS-DA model of HNF1A versus T2D/GCK; green triangle  =  HNF1A, red triangle  =  GCK and blue triangle  =  T2D. Q^2^ = 0.518 using three PLS-components in a valid model (Q^2^Y = 0.52).

We then tested if the metabolic profiles of the MODY subtypes, HNF1A-MODY and GCK-MODY, were different from T2D subjects in a three-class PLS-DA model. The model with low Q^2^ proved to be non-valid in a permutation test (Table S1). However, the HNF1A-MODY group was well separated from the two other groups in the score plot (Figure S3), and consequently a two-class model was built having merged the GCK-MODY and T2D groups. This model tested valid with Q^2^ above 0.5 using three components (Figure 1, Table S1). We examined the models for possible confounding effects from age, gender, BMI and family membership on the class prediction, but the valid models did not show any separation trends between the classes based on these factors.

**Figure 2:**
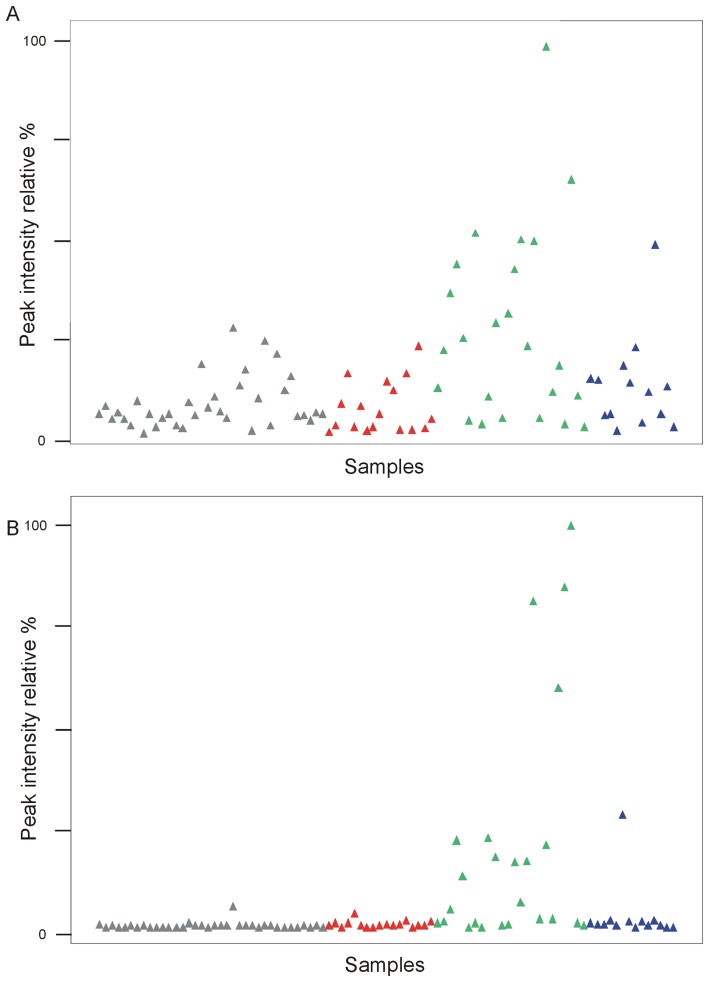
Scatter diagrams of betaine (A) and glucose adduct (B) ESI^+^-MS peak intensities in detected samples; grey triangle  =  control, green triangle  =  HNF1A, red triangle  =  GCK and blue triangle  =  T2D. Intensity of the signal is plotted as a constant sum normalized value.

In an attempt to look for interesting MS peak variables that contributed to the separation of the HNF1A-MODY group from the merged GCK-MODY and diet treated T2D groups, the S-plot from a two-class O-PLS-DA model (data not shown) was examined. Some of the MS peaks were singled out as the most important contributors to the HNF1A-MODY class separation. However, looking at the MS raw data it appeared the selected peaks had very high intensity levels in several of the HNF1A-MODY subjects, but their levels were at the level of the controls and the other diabetic groups in the rest of the HNF1A-MODY subjects (Figure 2). Thus, the clear separation of HNF1A-MODY subgroup from the other diabetic subgroups in the two-class model could not reveal any significant individual metabolic markers across the entire subgroup.

The two peaks with the highest contributions to the HNF1A-MODY subgroup separation were chosen for closer examination, and the corresponding metabolites were confirmed by comparison of accurate mass, retention time, co-elution, and MS/MS mass spectra to those of authentic standards. They were betaine (m/z 118.086), ESI^+^) and glucose (m/z 219.016, ESI^+^) (Figure 2). The high betaine and glucose levels were not directly correlated with each other in more than two subjects (Figure S4). The high glucose and betaine levels did not show any strong correlations when plotted against the clinical parameters measured (HbA1c, fasting blood glucose, BMI or duration of disease). There was however a correlation between betaine levels and gender. A plot of betaine peak intensity levels for the three diabetic subgroups showed that the HNF1A-MODY subjects with abnormal betaine levels were all female (Figure S5). However, this observation could be a coincidence considering the low number of subjects in the HNF1A-MODY group (n = 24), a majority of whom were female. The four non-diabetic HNF1A-MODY mutation carriers had some of the lowest betaine levels observed in this study, and when betaine levels were plotted against fasting plasma glucose levels for all HNF1A-MODY subjects, the four mutation carriers clustered together (Figure S6).

### Metabolic profiling of diabetic subjects by ^1^H nuclear magnetic resonance spectroscopy (NMR)

Similar to the modelling process of MS data, multivariate models were acquired for NMR data. The glucose regions of the NMR spectra had to be removed due to very high urine glucose levels in several of the HNF1A-MODY subjects. The processed NMR spectra were modelled with PLS-DA to discriminate the diabetic subgroups (Figure S7). The PLS-DA model showed reasonable separation of the three diabetic subgroups, but the model was not valid (Q^2^ = 0.06). In addition, the model was not improved when T2D and GCK-MODY groups were merged into one class of a two-class model. Examination of the loading plot of the PLS-DA model (data not shown) identified the NMR markers that contributed to the separation of HNF1A-MODY subjects from the other diabetic subgroups mainly as amino acids.

The changes in amino acids were then examined more closely using targeted profiling of the NMR spectra to obtain relative quantification of alanine, glycine, histidine, lysine, methionine, phenylalanine, threonine, tryptophan, tyrosine and valine. The HNF1A-MODY subjects showed significant increase in valine compared to GCK-MODY and T2D subjects, significant decrease in threonine compared to GCK-MODY subjects and significant increase in glycine compared to T2D subjects (Table S2).

### Validation of Signals found in Discovery Study

#### Urinary Glucose Analysis

Urinary glucose was measured in subjects with HNF1A-MODY, GCK-MODY and T2D. Results are shown in Table 2. Given that subjects with HNF1A-MODY are known to have a low renal threshold for glucose, we calculated both urinary glucose/creatinine (UG/Cr) ratio and plasma glucose to urinary glucose/creatinine ratio PG/(UG/Cr). These measures showed a significant difference across the three groups, with the least urinary glucose found in those with GCK-MODY and the most in those with HNF1A-MODY. Similarly the largest PG/(UG/Cr) ratio was found in those with GCK-MODY and the lowest with HNF1A-MODY. Subjects with T2D had intermediate values. This is in keeping with previous observations of serum 1,5AG levels, which are correlated with urine glucose levels [12].

**Table 2:** Follow up of glucose, amino acid and betaine signals.

	HNF1A-MODY	GCK-MODY	T2D	p	C statistic HNF1A vs T2D
**Urine Glucose**	n = 27	n = 17	n = 158		
Urinary glucose/Cr ratio (mmol/mmol)	0.67(1.84)	0.039(0.024)	0.13(1.88)	*0.01	0.58
Plasma glucose/Urinary glucose ratio (mmol/mmol)	0.73(5.87)	12.41(9.47)	6.92(19.14)	*0.04	0.63
Plasma glucose/(Urinary glucose/ creatinine ratio) mmol/ (mmol/mmol)	13.1 (138.3)	178. 9 (100.3)	72.0 (174.7)	*0.01	0.55
**Amino Acids**	n = 22		n = 22-		
Valine/Cr ratio (µmol/mmol)	0.35 (0.62)		0.67 (0.92)	0.08	
Alanine/Cr ratio (µmol/mmol)	2.00 (3.80)		4.79 (4.61)	0.18	
**One-Carbon compounds**	n = 39		N = 80		
Glycine/Cr ratio (µmol/mmol)	93.6 (73.6)		88.1 (68.0)	0.87	
Urinary betaine/Cr ratio (µmol/mmol)	19.5 (28.2)		34.9 (52.9)	<0.001	0.70
Sarcosine/Cr ratio (µmol/mmol)	0.21 (0.25)		0.27 (0.24)	0.03	0.63
Choline/Cr ratio (µmol/mmol)	2.75 (2.59)		4.00 (3.43)	0.003	0.67

Data are median (IQR). P values compare HNF1A and T2D using Mann Whitney U test except for * where comparisons between all 3 groups are calculated by Kruskal-Wallis test.

We then examined whether UG/Cr or PG/(UG/Cr) could be used to discriminate HNF1A-MODY from young-onset T2D using Receiver Operating Characteristic (ROC) Curve analysis. The area under the ROC curve for both these two parameters was <0.6, showing that features based on urine glucose do not provide clinically useful discrimination between HNF1A-MODY and T2D.

#### Urinary Amino Acid Analysis

Once matched for urinary glucose levels, there was no difference observed in the levels of the urinary amino acids valine and glycine, which were noted in the Discovery study to be increased in HNF1A-MODY subjects (Table 2).

#### Urinary Betaine Analysis

Betaine/Cr ratio was noted to be high in the urine of HNF1A-MODY subjects in the Discovery Study compared to Diet-controlled T2D and GCK-MODY. On quantifying the extended dataset, however, the opposite finding was noted with markedly higher betaine noted in the T2D group. Two close metabolites of betaine were also higher in the T2D group: Choline, which is a precursor of betaine, and sarcosine, a product of betaine demethylation. Urinary betaine/Cr levels were correlated with fasting glucose (p = 2×10^−5^), urine glucose (p = 3.5×10^−4^) and HbA1c (1×10^−4^). Linear regression showed that the significant determinants of urinary betaine/Cr were urinary glucose (p = 0.021) and type of diabetes (p = 0.038). After controlling for urinary glucose levels, fasting glucose and HbA1c did not significantly affect betaine/Cr ratio.

#### Biomarkers in combination with clinical features

It is likely that combinations of clinical features and biomarkers will be used in clinical algorithms to identify those at high risk of MODY. Linear regression modelling in the HNF1A-MODY and T2D cases from the extended dataset showed that (young) age of onset of diabetes was the most important predictor of HNF1A-MODY (P<0.001), with urinary glucose/Cr ratio having a more modest effect (p = 0.03).

## Discussion

We designed this study to try to identify urinary markers which might be useful in the clinical discrimination of diabetes subtypes in young adults; a group in whom many are missing the opportunities offered by accurate molecular diagnosis. This study has the advantage of a biology agnostic design to identify metabolic markers associated with monogenic subtypes without making prior assumptions about candidacy.

PLS-DA models were used explore the metabolic discrimination between normoglycaemic controls and the various diabetic subtypes. The models showed that compared to the normoglycaemic controls all the diabetic subjects were metabolically ‘similar’ and had to be treated as one class to make a valid discrimination model against the controls. The four non-diabetic *HNF1A* mutation carriers showed more features of the diabetic subjects than the normoglycaemic controls despite their fasting blood glucose and HbA1c values being within the normal range. This is in agreement with previous studies on non-diabetic *HNF1A* mutation carriers which demonstrated that many aspects of the HNF1A-MODY diabetic phenotype, such as β-cell deficiency and low renal threshold for glucose, are already present in the mutation carriers before the development of frank diabetes [21].

The diabetic subtype discrimination model showed that the HNF1A metabolic profile differs sufficiently from the other two subtypes to make a valid model. However, the attempt to classify GCK and T2D separately failed. This probably reflects that the diet-controlled T2D subjects used in the discovery part of the study were metabolically closer to the GCK-MODY subjects than a more typical T2D subject (and this may be a limitation of the design of this study in terms of finding markers that discriminate T2D and GCK-MODY).

Using the UPLC-MS methodology, we identified two peaks that were associated with HNF1A-MODY cases rather than T2D and GCK-MODY subjects. Firstly they had increased urinary glucose levels. This was not surprising as low renal threshold is seen clinically in this form of diabetes and attributed to reduced expression of the low affinity/high-capacity glucose co-transporter (*SGLT2*) which is regulated by HNF1A [22]. In this study we have examined for the first time whether parameters derived directly from urinary glucose measurement can be used as a simple diagnostic screen for HNF1A-MODY cases. Although a significant difference is observed, this is not of high discriminative accuracy as demonstrated by a ROC curve derived C-statistic of <0.6.

The MS study also identified increased urinary betaine levels in the HNF1A-MODY subjects. We were unable to confirm this in the extended study – in fact we observed that betaine excretion was significantly higher in individuals with T2D. Betaine is important in mammalian physiology in two main ways [23]: firstly it is involved in metabolism of one-carbon compounds by acting as the donor of a methyl group in the conversion of homocysteine to methionine and secondly it is one of the major osmolytes utilised in the kidney and other tissues. Urinary betaine excretion is known to be increased in diabetic individuals [24] and in those who have metabolic syndrome [25].

The increased urinary betaine excretion seen in those with metabolic syndrome means we would expect that subjects with T2D would have higher urine betaine as we saw in the extended study. The initial observation was thus likely to be spurious and due to differences in the T2D groups used in the two arms of the study. The diet-controlled T2D subjects selected for the discovery part of the study had lower fasting glucose and HbA1c levels than the more typical T2D group used in the extended study (both p<0.03 between discovery and extended study groups). Betaine excretion has been previously reported to be correlated with fasting glucose levels [24] and from our results appears to be partly driven by glycosuria (which is itself correlated with fasting glucose and HbA1c). This might be expected to increase betaine excretion in subjects such as the HNF1A-MODY cases who have a low renal threshold for glucose. Interestingly our observation that urine glucose excretion is correlated with urinary betaine has recently been reported by another group [26], the mechanisms of this warrant further investigation.

Alterations in serum and urinary amino acids are seen in the *Hnf1a*−/− mouse and have been investigated previously as candidate biomarkers for HNF1A-MODY [8], [16]. In the study by Bingham et al [8], aminoaciduria was seen in other forms of diabetes as well as HNF1A-MODY and was thought to be driven by the presence of glycosuria. We matched for urine glucose in order to establish definitively whether there were any differences in urinary amino acids between T2D and HNF1A-MODY that were additional to the effects of glycosuria. No differences were seen and thus we conclude that urinary amino acid profile cannot be used as a marker for HNF1A-MODY.

In conclusion, the overall findings of this study were that there were no robust differences found on metabolic profiling of young-adults with different diabetes subtypes that would aid clinical discrimination of these subtypes and identify those in whom genetic testing might be beneficial. We did however confirm the expected difference of increased urinary glucose in HNF1A-MODY highlighting that the numbers of cases included and the study design was adequate to identify differences between the subgroups. However, the T2D group, selected because they were diet controlled and thus on few medications and without complications of diabetes, was too metabolically similar to the GCK–MODY group to allow good separation.

Our study has implications for metabolic profiling in complex metabolic disorders such as T2D, as despite using homogenous subgroups with different underlying pathophysiologies, we have failed to detect urinary metabolic differences which translate into robust biomarkers. Alternative omic approaches such as peptide or protemomics might be more effective in identifying robust biomarkers for diabetes subtype discrimination.

## Materials and Methods

### Ethics Statement

The study was performed according to the latest version of the Helsinki Declaration. Ethical approval was obtained from the Oxfordshire local research ethics committee and all subjects gave written informed consent prior to participation.

### Subjects

For the initial Discovery study we included 14 cases of HNF1A-MODY, 17 of GCK-MODY, 14 cases of diet controlled young-onset T2D (diagnosed up to 45 years of age) and 34 non-diabetic individuals (Table 1). Due to the lower prevalence of HNF4A-MODY compared with GCK and HNF1A-MODY we were unable to recruit individuals with *HNF4A* mutations for this study [2]. Subjects for the extended study were selected from a larger pool of subjects as detailed below. The T2D subjects used in the extended study were independent of those used in the Discovery study, and represented a more typical mixture of T2D cases (longer duration, treated with diet, oral agents or insulin). The HNF1A-MODY cases in the extended study included as a subset those who took part in the Discovery study.

Subjects were ascertained from Oxfordshire, UK and those with MODY from Oxfordshire and other counties in the South of England. The MODY subjects comprised subjects with known pathogenic mutation (confirmed by sequencing in a certified UK diagnostic centre) in either *HNF1A* or *GCK*. The individuals with MODY were recruited from patients known to our clinical service, family members of those patients or patients from other hospitals identified by the Genetic Diabetes Nurse network. One HNF1A-MODY had impaired glucose tolerance (IGT) and 3 were normoglycaemic; the remainder had diabetes. Oral glucose tolerance test (OGTT) data at the time of sampling were available on the non-diabetic HNF1A-MODY mutation carriers to confirm diabetic status. Subjects with *GCK* mutations are affected with lifelong fasting hyperglycaemia.

The T2D subjects were selected from the Young Diabetes in Oxford study, comprising subjects diagnosed with diabetes ≤45 years of age, C-peptide positive and with negative GAD antibodies. Individuals selected for the Discovery study were all diet treated, while those in the extended study were on various treatments (see Table 1). Subjects did not meet clinical criteria for MODY diagnostic testing [5] or had been tested and were negative for mutations in *HNF1A/HNF4A* and/or *GCK*.

Non-diabetic subjects (ND, n = 34) in the Discovery study were recruited from the Oxford Biobank (http://www.oxfordbrc.org/research/chronic-disease-cohorts/102/), a population-based collection of healthy adults aged 30–50 recruited for translational medicine studies. Normoglycaemia was defined as fasting glucose <6.0 mmol/l and no history of a diagnosis of diabetes. All subjects were of European ethnicity.

### Sample Collection and Preparation

An early morning urine sample was supplied by each subject. The urine was centrifuged and the supernatant separated and stored at −80°C. Analysis of urinary glucose and creatinine as well as a paired sample for fasting plasma glucose, HbA1c and serum creatinine was performed in the Department of Biochemistry at the John Radcliffe Hospital, Oxford using standard analysis methods. The samples were prepared for UPLC-MS and NMR analyses according to sampling procedure in the supplementary online methods (Text S1).

### UPLC-MS

The UPLC Q-Tof MS system comprised an AQUITY UPLC hyphenated to a Q-TOF Premier mass spectrometer (Waters, Milford, USA) (for details see supplementary online methods, Text S1). The identification process of important metabolites was performed by accurate mass and MS/MS measurements and confirmed by co-elution with commercially purchased standards.

### ^1^H NMR spectroscopy

One-dimensional **^1^**H NMR spectra were acquired on a Bruker DRX 600 spectrometer (Bruker BioSpin, Rheinstetten, Germany) (for details see supplementary online methods, Text S1). Assignments of metabolites were based on literature values. The targeted profiling approach in the Chenomx NMR Suite v7.0 (Chenomx Inc., Alberta, Canada) was used to relatively quantify amino acids.

### Data processing

Control urine samples were used to check the reproducibility of MS and NMR data acquisition across well plates. MassLynx v4.0 (Waters, Milford, USA) was used to convert the centroid UPLC-MS data files to NetCDF format for further processing. XCMS [27] software was used for peak matching, non-linear retention time alignment and quantitation of mass spectral ion intensities across all NetCDF mass spectral files. The method is described in detail in the Supplementary online methods. Normalised data sets were constructed using constant sum normalisation. The NMR spectra were phase and baseline corrected and chemical shift aligned to the internal standard. Spectral regions corresponding to water were removed. Glucose regions were also removed due to high urine glucose levels in many of the HNF1A-MODY samples. The full resolution NMR spectra were normalized using probabilistic quotient normalisation [28].

### Data analysis

The MS peaks present in blank water samples were removed across all spectra, and all models were calculated with univariate scaled data. All NMR models used Pareto scaled data. Principal component analysis (PCA) of the MS and NMR data was used to check for outliers and trends due to gender, age, and family. Partial least squares discriminant analysis (PLS-DA) was used to model the discrimination between the HNF1A, GCK, T2D and ND groups. Cross-validation with seven cross-validation groups was used to determine the number of components. The validity of the PLS-DA models was assessed using a response permutation test with 200 permutations, and Q^2^>0.5 was considered to be a good model. O-PLS-DA model S-plot [29] was used to look for MS peaks that might contribute significantly to the separation of the diabetic subclasses.

Individual urine amino acids determined by ^1^H NMR spectroscopy were compared across the groups of diabetic individuals by unpaired t-tests.

### Quantification of urinary Valine and Glycine

A subset of 22 HNF1A-MODY and 22 T2D subjects were selected for analysis. Samples were matched for urine glucose, to control for the known effect of glycosuria as a cause of aminoaciduria [8]. Analysis was performed in the Biochemistry lab at the John Radcliffe Hospital, Oxford. Samples for amino acid analysis underwent pre-column derivatisation using AccQ·Fluor reagent (Waters, Milford, USA). Briefly, internals standards (methylphenylalanine and methionine sulfone, Sigma) were added to each specimen. Proteins were precipitated by the addition of acetonitrile and isolated by centrifugation. Supernatants were subsequently derivatised using AccQ·Fluor reagent. Amino acid derivatives were separated on a Waters ACQUITY UPLC system (Waters, Milford, USA) with UV absorbance measured at 260 nm. Gradient chromatography was employed using AccQ·Tag Ultra mobile phases (AccQ·Tag Ultra eluent A and AccQ·Tag Ultra eluent B), flow rate of 0.6 ml/min, injection volume of 1 µl, ACQUITY UPLC BEH C18 column (1.7 µm 2.1×150 mm), column temperature of 60°C. Calibration was performed using an amino acid standard solution (Sigma) and analyses were verified using in-house quality control material. Data analysis was performed using Empower 2 software (Waters, Milford, USA).

### Quantification of betaine, related methylamines and selected amino acids

Thirty-nine HNF1A-MODY and 80 T2D subjects were selected for analysis. Samples were analysed by Bevital laboratories, Bergen, Norway using high-throughput systems [23]. Betaine and a number of close metabolites in the one-carbon pathway were selected for analysis: total homocysteine, methylmalonic acid, cysteine, methionine, serine, glycine, cystathionine, kynurenine (analysed by gas chromatography/mass spectrometry); sarcosine and tryptophan (gas chromatography/tandem mass spectrometry) and choline, betaine, dimethylglycine, arginine and asymmetric/symmetric dimethylarginine (by liquid chromatography/tandem mass spectrometry).

### Statistical Analysis for the amino acid and one-carbon validations

Differences between medians were calculated using Mann Whitney U or Kruskal Wallis tests. Statistical analysis was performed in SPSS v18 and p<0.05 was considered significant.

## Supporting Information

Figure S1 **Typical base peak MS chromatograms obtained from urine of: A) T2D; B) GCK; C) HNF1A and D) healthy controls, scanned by ESI^+^.**
(TIF)

Figure S2 **Score plot of a 2-class PLS-DA model of the control group versus the three diabetic subgroups together; Grey triangle  =  control, green triangle  =  HNF1A, red triangle  =  GCK and blue triangle  =  T2D.** Q2 = 0.55 using two PLS components in a valid model. The samples in red boxes are three HNF1A non-diabetic mutation carriers; the blue box sample is a HNF1A IGT mutation carrier.
(TIF)

Figure S3 **Score plot of a three-class PLS-DA model of the three diabetic subgroups using ESI^+^-MS data.** Q2 = 0.011 for the first two components in a non-valid model. Grey triangle  =  control, green triangle  =  HNF1A, red triangle  =  GCK and blue triangle  =  T2D.
(TIF)

Figure S4 **Plot of betaine versus glucose ESI^+^-MS peak intensities from all diabetic subjects.** Intensity of the signal is plotted as a constant sum normalized value. Grey triangle  =  control, green triangle  =  HNF1A, red triangle  =  GCK and blue triangle  =  T2D. Regression coefficient (R2)  = 0.1662.
(TIF)

Figure S5 **Profile of betaine excretion from ESI^+^-MS data stratified by diabetes subtype and gender.** Intensity of the signal is plotted as a constant sum normalized value (diamond: male; red cross: female).
(TIF)

Figure S6 **Plot of betaine ESI^+^-MS peak intensity versus measured fasting glucose levels of all HNF1A-MODY subjects.** The samples in red boxes are three non-diabetic *HNF1A* mutation carriers; the blue box sample is an *HNF1A* mutation carrier with impaired glucose tolerance.
(TIF)

Figure S7 **Score plot of a three-class PLS-DA model of the three diabetic subgroups using NMR data.** Q2 = 0.06 for the first two components in a non-valid model. Grey triangle  =  control, green triangle  =  HNF1A, red triangle  =  GCK and blue triangle  =  T2D.
(TIF)

Text S1 **Online supplementary methods.**
(DOC)

Table S1 **Q^2^Y (goodness of fit) for PLS-DA models.**
(DOC)

Table S2 **Amino acid concentrations in urine measured by ^1^H NMR in the diabetic subgroups.**
(DOC)

## References

[1] Kropff J, Selwood MP, McCarthy MI, Farmer AJ, Owen KR (2011) Prevalence of monogenic diabetes in young adults: a community-based, cross-sectional study in Oxfordshire, UK. Diabetologia 54: 1261–1263. 21350841 10.1007/s00125-011-2090-z

[2] Shields BM, Hicks S, Shepherd MH, Colclough K, Hattersley AT, et al (2010) Maturity-onset diabetes of the young (MODY): how many cases are we missing? Diabetologia 53: 2504–2508. 20499044 10.1007/s00125-010-1799-4

[3] Murphy R, Ellard S, Hattersley AT (2008) Clinical implications of a molecular genetic classification of monogenic beta-cell diabetes. Nat Clin Pract Endocrinol Metab 4: 200–213. 18301398 10.1038/ncpendmet0778

[4] Pearson ER, Starkey BJ, Powell RJ, Gribble FM, Clark PM, et al (2003) Genetic cause of hyperglycaemia and response to treatment in diabetes. Lancet 362: 1275–1281. 14575972 10.1016/S0140-6736(03)14571-0

[5] Ellard S, Bellanne-Chantelot C, Hattersley AT (2008) Best practice guidelines for the molecular genetic diagnosis of maturity-onset diabetes of the young. Diabetologia 51: 546–553. 18297260 10.1007/s00125-008-0942-y PMC2270360

[6] Thanabalasingham G, Shah N, Vaxillaire M, Hansen T, Tuomi T, et al (2011) A large multi-centre European study validates high-sensitivity C-reactive protein (hsCRP) as a clinical biomarker for the diagnosis of diabetes subtypes. Diabetologia 54: 2801–2810. 21814873 10.1007/s00125-011-2261-y

[7] Servitja JM, Pignatelli M, Maestro MA, Cardalda C, Boj SF, et al (2009) Hnf1alpha (MODY3) controls tissue-specific transcriptional programs and exerts opposed effects on cell growth in pancreatic islets and liver. Mol Cell Biol 29: 2945–2959. 19289501 10.1128/MCB.01389-08 PMC2682018

[8] Bingham C, Ellard S, Nicholls AJ, Pennock CA, Allen J, et al (2001) The generalized aminoaciduria seen in patients with hepatocyte nuclear factor-1alpha mutations is a feature of all patients with diabetes and is associated with glucosuria. Diabetes 50: 2047–2052. 11522670 10.2337/diabetes.50.9.2047

[9] Cervin C, Axler O, Holmkvist J, Almgren P, Rantala E, et al (2010) An investigation of serum concentration of apoM as a potential MODY3 marker using a novel ELISA. J Intern Med 267: 316–321. 19754856 10.1111/j.1365-2796.2009.02145.x

[10] Karlsson E, Shaat N, Groop L (2008) Can complement factors 5 and 8 and transthyretin be used as biomarkers for MODY 1 (HNF4A-MODY) and MODY 3 (HNF1A-MODY)? Diabet Med 25: 788–791. 18513302 10.1111/j.1464-5491.2008.02467.x

[11] Owen KR, Thanabalasingham G, James TJ, Karpe F, Farmer AJ, et al (2010) Assessment of high-sensitivity C-reactive protein levels as diagnostic discriminator of maturity-onset diabetes of the young due to HNF1A mutations. Diabetes Care 33: 1919–1924. 20724646 10.2337/dc10-0288 PMC2928334

[12] Pal A, Farmer AJ, Dudley C, Selwood MP, Barrow BA, et al (2010) Evaluation of serum 1,5 anhydroglucitol levels as a clinical test to differentiate subtypes of diabetes. Diabetes Care 33: 252–257. 19933992 10.2337/dc09-1246 PMC2809258

[13] Richter S, Shih DQ, Pearson ER, Wolfrum C, Fajans SS, et al (2003) Regulation of apolipoprotein M gene expression by MODY3 gene hepatocyte nuclear factor-1alpha: haploinsufficiency is associated with reduced serum apolipoprotein M levels. Diabetes 52: 2989–2995. 14633861 10.2337/diabetes.52.12.2989

[14] Skupien J, Gorczynska-Kosiorz S, Klupa T, Wanic K, Button EA, et al (2008) Clinical application of 1,5-anhydroglucitol measurements in patients with hepatocyte nuclear factor-1alpha maturity-onset diabetes of the young. Diabetes Care 31: 1496–1501. 18492944 10.2337/dc07-2334 PMC2494661

[15] Skupien J, Kepka G, Gorczynska-Kosiorz S, Gebska A, Klupa T, et al (2007) Evaluation of Apolipoprotein M Serum Concentration as a Biomarker of HNF-1alpha MODY. Rev Diabet Stud 4: 231–235. 18338076 10.1900/RDS.2007.4.231 PMC2270407

[16] Stride A, Pearson ER, Brown A, Gooding K, Castleden HA, et al (2004) Serum amino acids in patients with mutations in the hepatocyte nuclear factor-1 alpha gene. Diabet Med 21: 928–930. 15270800 10.1111/j.1464-5491.2004.01107.x

[17] McDonald TJ, Shields BM, Lawry J, Owen KR, Gloyn AL, et al (2011) High-Sensitivity CRP Discriminates HNF1A-MODY From Other Subtypes of Diabetes. Diabetes Care. 34: 1860–1862. 10.2337/dc11-0323 PMC3142017 21700917

[18] Bonzo JA, Patterson AD, Krausz KW, Gonzalez FJ (2010) Metabolomics identifies novel Hnf1alpha-dependent physiological pathways in vivo. Mol Endocrinol 24: 2343–2355. 20943816 10.1210/me.2010-0130 PMC2999475

[19] Wang TJ, Larson MG, Vasan RS, Cheng S, Rhee EP, et al (2011) Metabolite profiles and the risk of developing diabetes. Nat Med 17: 448–453. 21423183 10.1038/nm.2307 PMC3126616

[20] Patterson AD, Bonzo JA, Li F, Krausz KW, Eichler GS, et al (2011) Metabolomics reveals attenuation of the SLC6A20 kidney transporter in nonhuman primate and mouse models of type 2 diabetes mellitus. J Biol Chem 286: 19511–19522. 21487016 10.1074/jbc.M111.221739 PMC3103330

[21] Stride A, Ellard S, Clark P, Shakespeare L, Salzmann M, et al (2005) Beta-cell dysfunction, insulin sensitivity, and glycosuria precede diabetes in hepatocyte nuclear factor-1alpha mutation carriers. Diabetes Care 28: 1751–1756. 15983330 10.2337/diacare.28.7.1751

[22] Pontoglio M, Prie D, Cheret C, Doyen A, Leroy C, et al (2000) HNF1alpha controls renal glucose reabsorption in mouse and man. EMBO Rep 1: 359–365. 11269503 10.1093/embo-reports/kvd071 PMC1083745

[23] Ueland PM (2011) Choline and betaine in health and disease. J Inherit Metab Dis 34: 3–15. 20446114 10.1007/s10545-010-9088-4

[24] Dellow WJ, Chambers ST, Lever M, Lunt H, Robson RA (1999) Elevated glycine betaine excretion in diabetes mellitus patients is associated with proximal tubular dysfunction and hyperglycemia. Diabetes Res Clin Pract 43: 91–99. 10221661 10.1016/s0168-8227(98)00115-6

[25] Lever M, George PM, Dellow WJ, Scott RS, Chambers ST (2005) Homocysteine, glycine betaine, and N, N-dimethylglycine in patients attending a lipid clinic. Metabolism 54: 1–14. 15562374 10.1016/j.metabol.2004.07.007

[26] Lever M, Slow S, McGregor DO, Dellow WJ, George PM, et al (2012) Variability of plasma and urine betaine in diabetes mellitus and its relationship to methionine load test responses: an observational study. Cardiovasc Diabetol 11: 34. 22510294 10.1186/1475-2840-11-34 PMC3395555

[27] Smith CA, Want EJ, O'Maille G, Abagyan R, Siuzdak G (2006) XCMS: processing mass spectrometry data for metabolite profiling using nonlinear peak alignment, matching, and identification. Anal Chem 78: 779–787. 16448051 10.1021/ac051437y

[28] Dieterle F, Ross A, Schlotterbeck G, Senn H (2006) Probabilistic quotient normalization as robust method to account for dilution of complex biological mixtures. Application in 1H NMR metabonomics. Anal Chem 78: 4281–4290. 16808434 10.1021/ac051632c

[29] Wiklund S, Johansson E, Sjostrom L, Mellerowicz EJ, Edlund U, et al (2008) Visualization of GC/TOF-MS-based metabolomics data for identification of biochemically interesting compounds using OPLS class models. Anal Chem 80: 115–122. 18027910 10.1021/ac0713510

