# Supplementary figures and images for: Metabolic Profiling in Maturity-Onset Diabetes of the Young (MODY) and Young Onset Type 2 Diabetes Fails to Detect Robust Urinary Biomarkers
Source: PLoS One. 2012 Jul 30;7(7):e40962. doi: 10.1371/journal.pone.0040962 (PMC3408469; doi:10.1371/journal.pone.0040962)

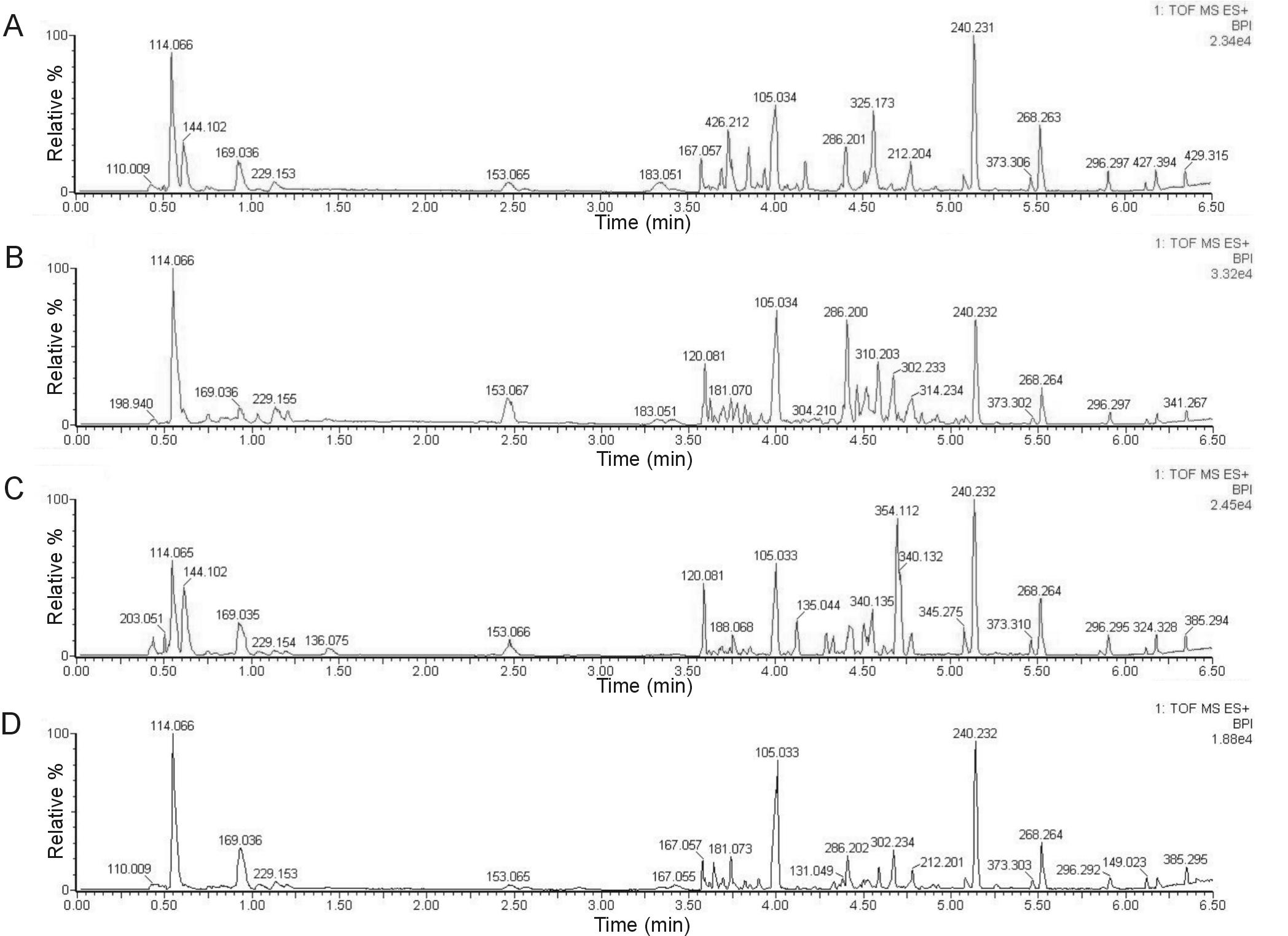

Supplement: Figure S1 — Typical base peak MS chromatograms obtained from urine of: A) T2D; B) GCK; C) HNF1A and D) healthy controls, scanned by ESI+. (TIF) [file pone.0040962.s001.tif]

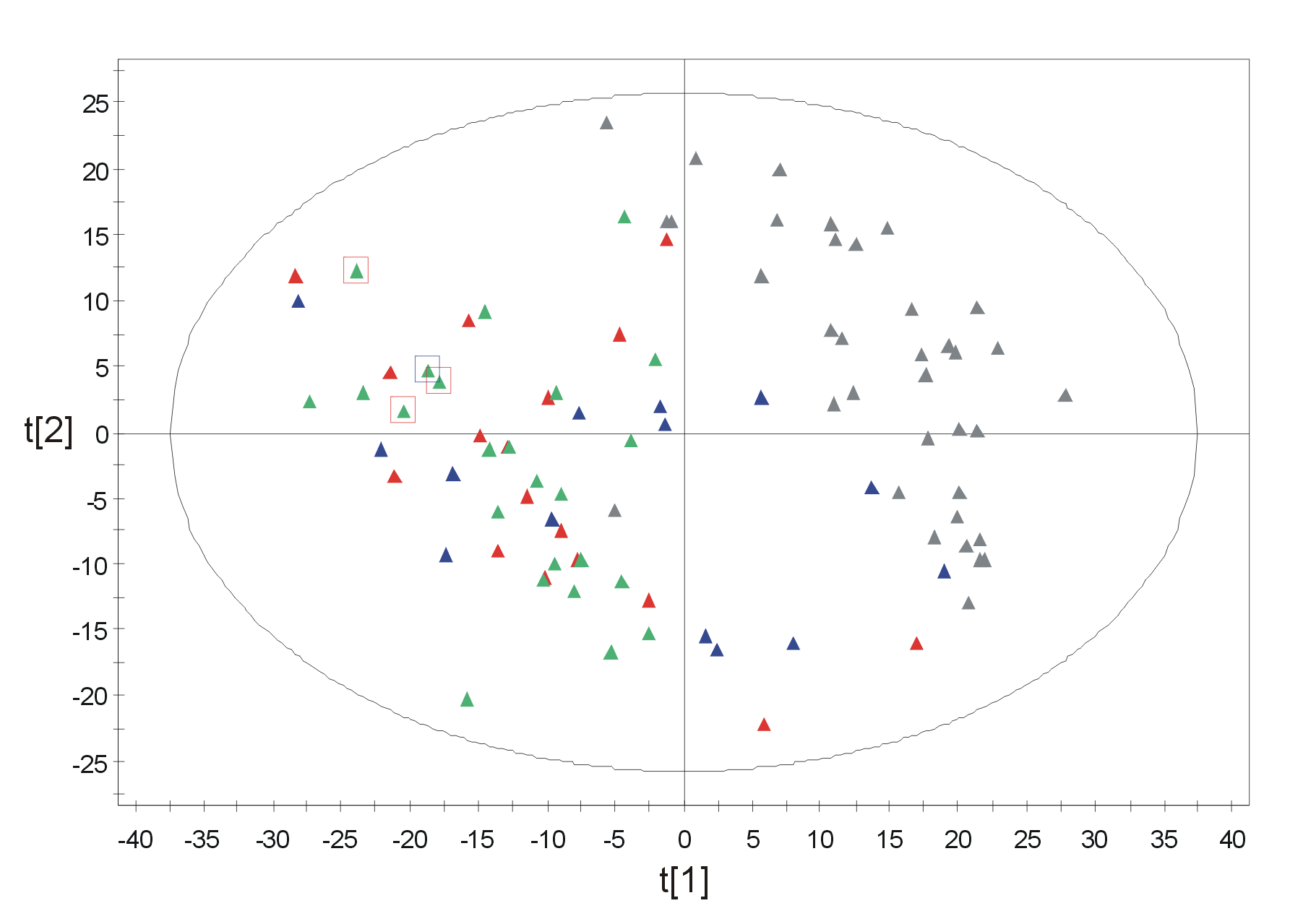

Supplement: Figure S2 — Score plot of a 2-class PLS-DA model of the control group versus the three diabetic subgroups together; Grey triangle = control, green triangle = HNF1A, red triangle = GCK and blue triangle = T2D. Q2 = 0.55 using two PLS components in a valid model. The samples in red boxes are three HNF1A non-diabetic mutation carriers; the blue box sample is a HNF1A IGT mutation carrier. (TIF) [file pone.0040962.s002.tif]

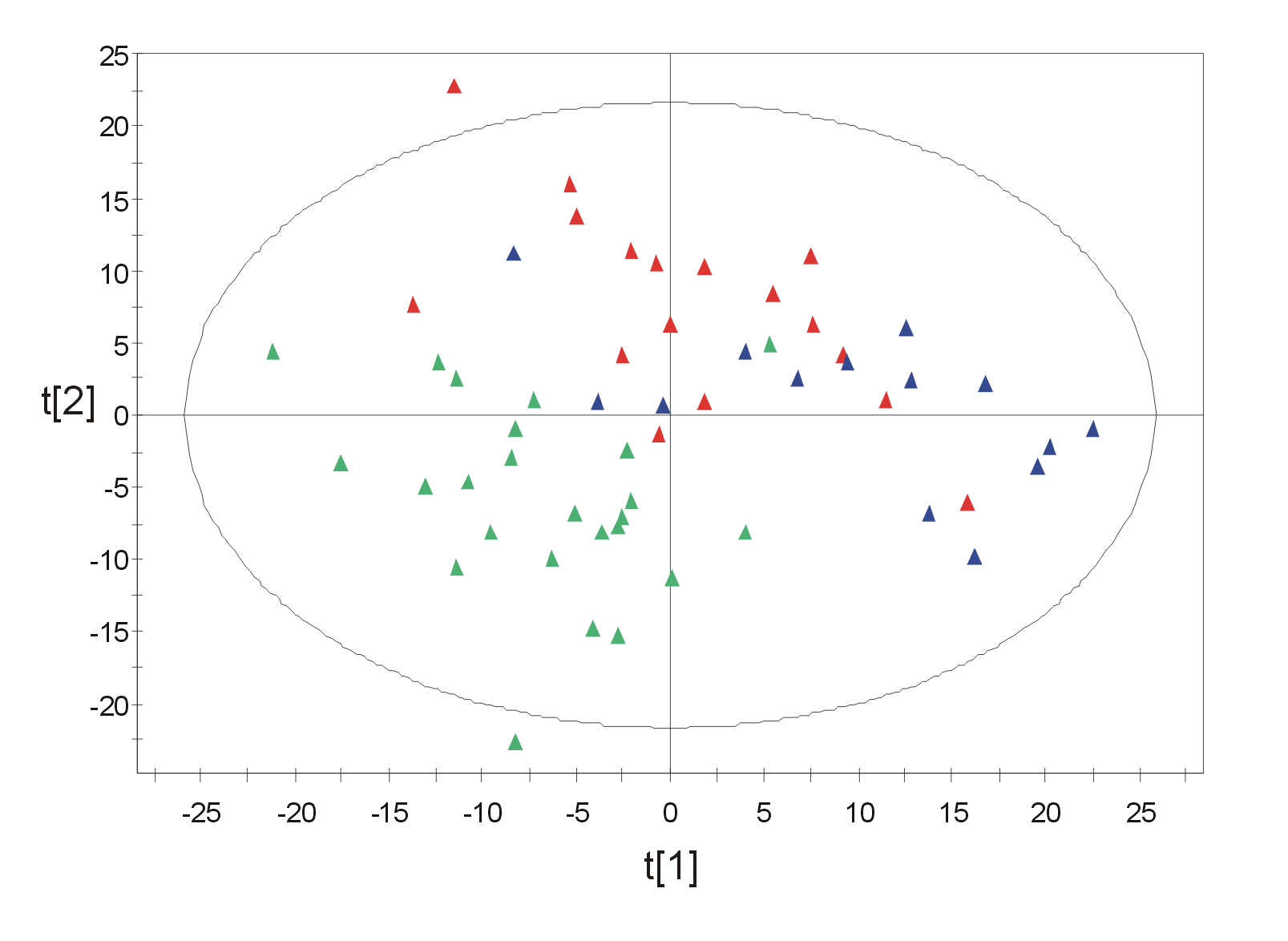

Supplement: Figure S3 — Score plot of a three-class PLS-DA model of the three diabetic subgroups using ESI+-MS data. Q2 = 0.011 for the first two components in a non-valid model. Grey triangle = control, green triangle = HNF1A, red triangle = GCK and blue triangle = T2D. (TIF) [file pone.0040962.s003.tif]

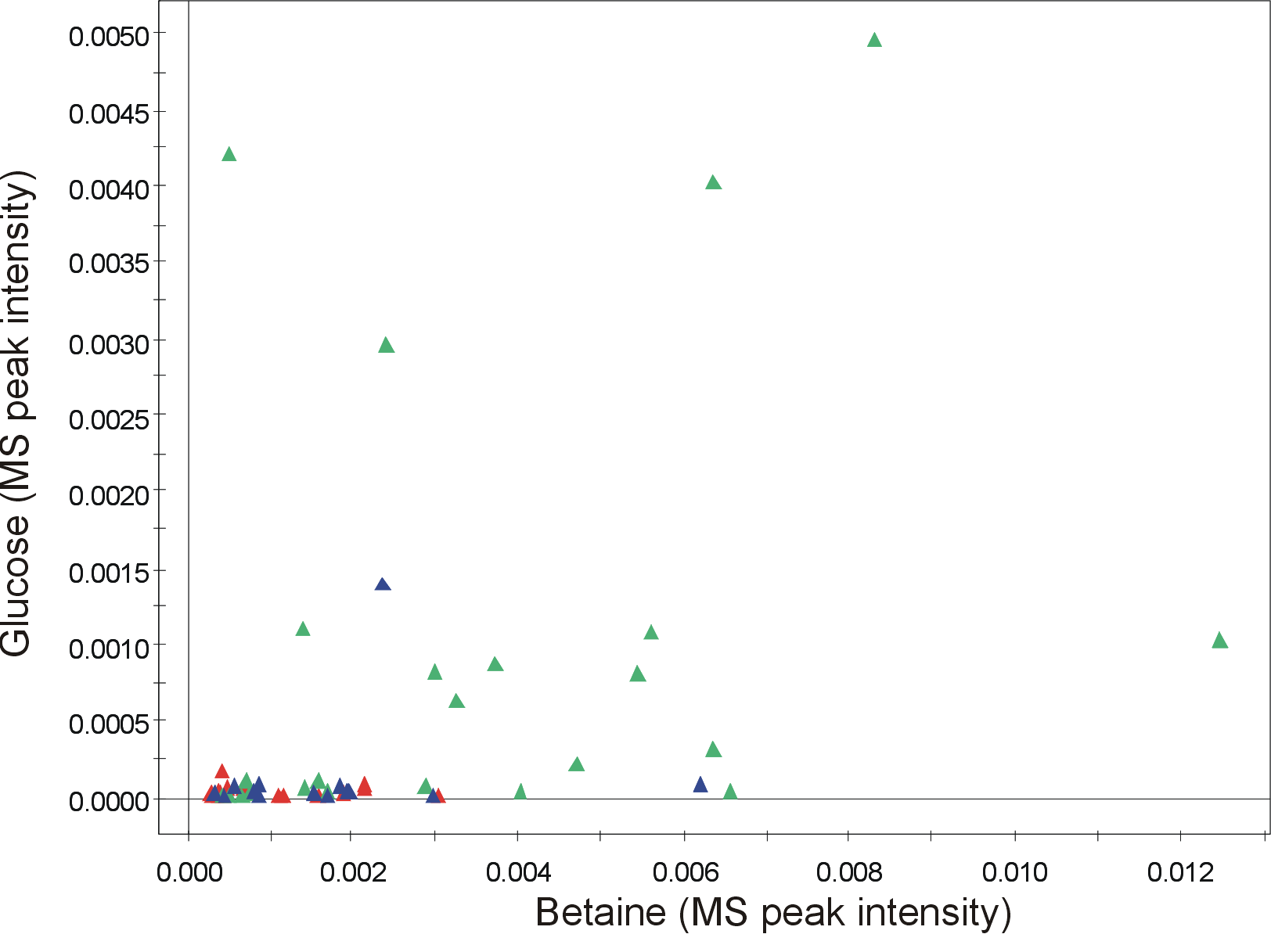

Supplement: Figure S4 — Plot of betaine versus glucose ESI+-MS peak intensities from all diabetic subjects. Intensity of the signal is plotted as a constant sum normalized value. Grey triangle = control, green triangle = HNF1A, red triangle = GCK and blue triangle = T2D. Regression coefficient (R2) = 0.1662. (TIF) [file pone.0040962.s004.tif]

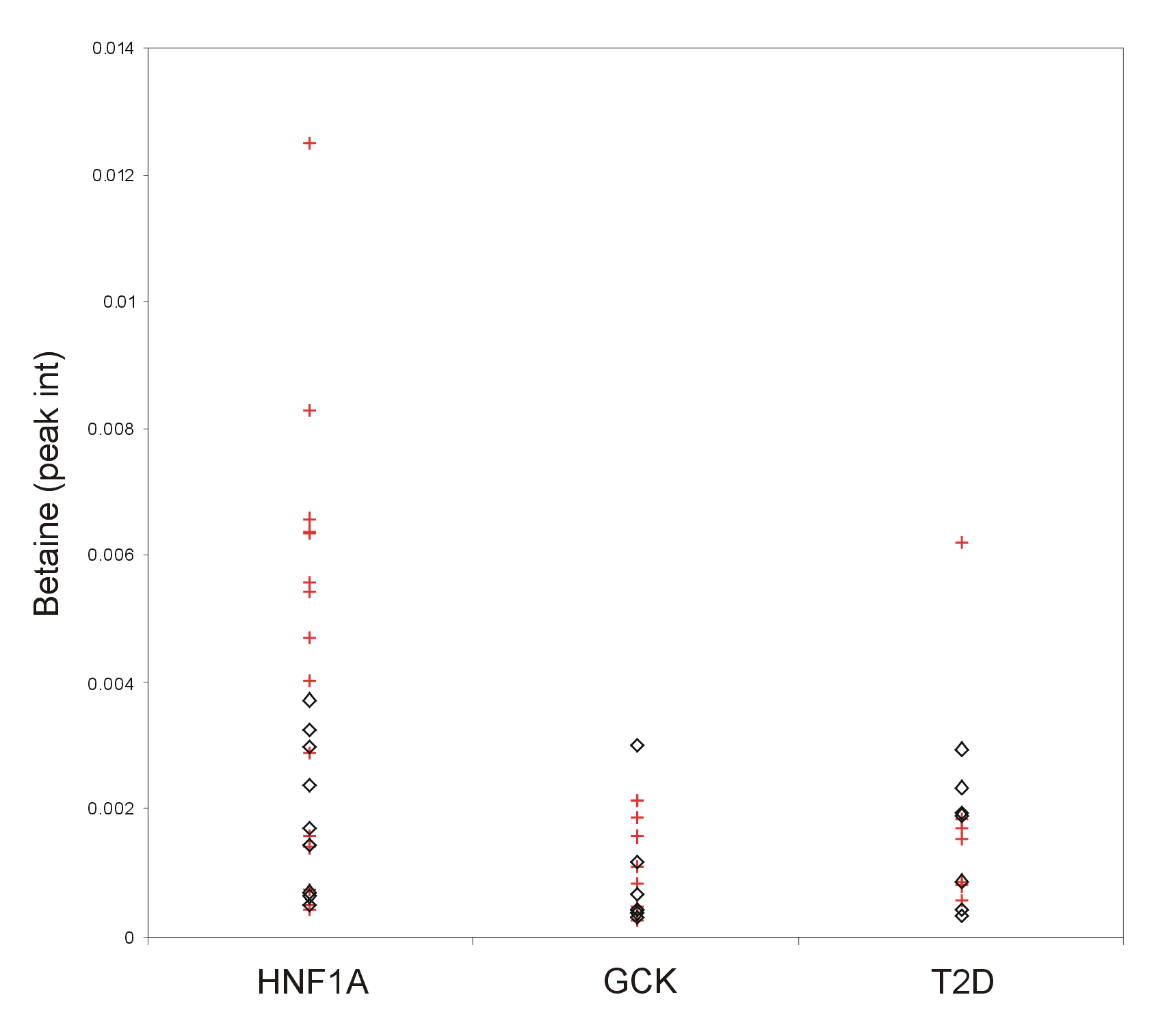

Supplement: Figure S5 — Profile of betaine excretion from ESI+-MS data stratified by diabetes subtype and gender. Intensity of the signal is plotted as a constant sum normalized value (diamond: male; red cross: female). (TIF) [file pone.0040962.s005.tif]

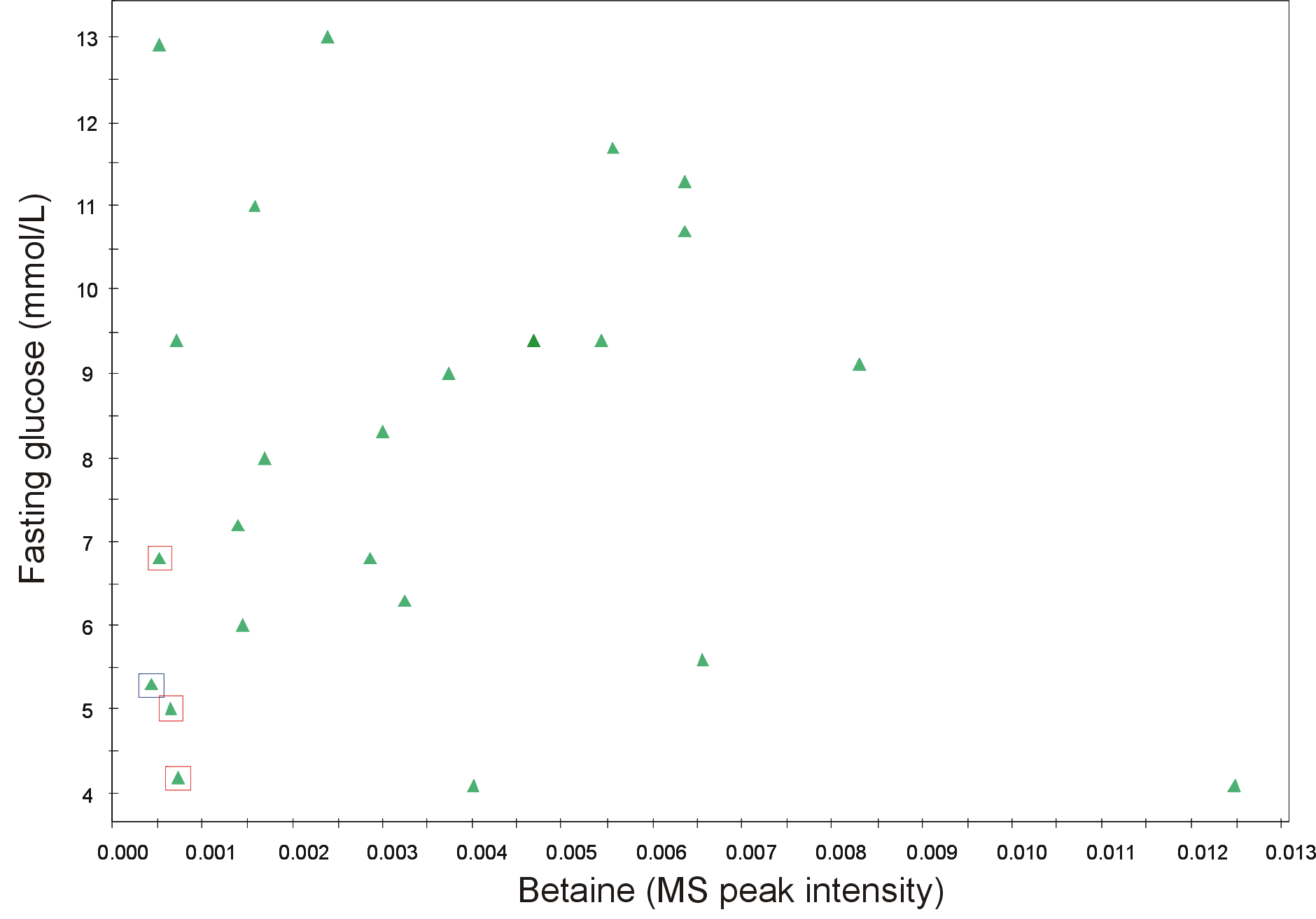

Supplement: Figure S6 — Plot of betaine ESI+-MS peak intensity versus measured fasting glucose levels of all HNF1A-MODY subjects. The samples in red boxes are three non-diabetic HNF1A mutation carriers; the blue box sample is an HNF1A mutation carrier with impaired glucose tolerance. (TIF) [file pone.0040962.s006.tif]

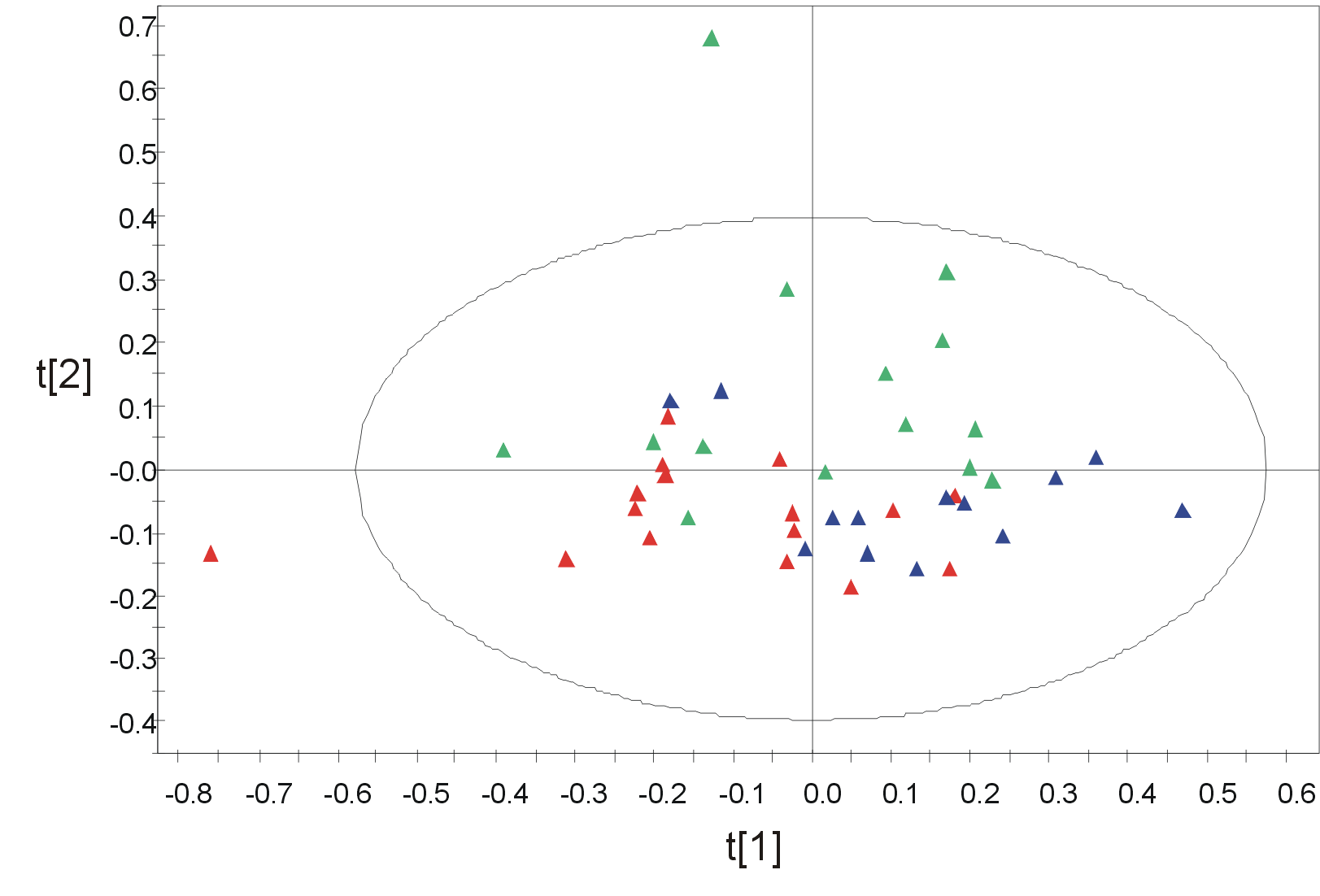

Supplement: Figure S7 — Score plot of a three-class PLS-DA model of the three diabetic subgroups using NMR data. Q2 = 0.06 for the first two components in a non-valid model. Grey triangle = control, green triangle = HNF1A, red triangle = GCK and blue triangle = T2D. (TIF) [file pone.0040962.s007.tif]
