# Supplementary material for: Metabolic Profiling in Maturity-Onset Diabetes of the Young (MODY) and Young Onset Type 2 Diabetes Fails to Detect Robust Urinary Biomarkers
Source: PLoS One. 2012 Jul 30;7(7):e40962. doi: 10.1371/journal.pone.0040962 (PMC3408469; doi:10.1371/journal.pone.0040962)
Supplement: Text S1 — Online supplementary methods. (DOC) [file pone.0040962.s008.doc]

**METABOLIC PROFILING IN MATURITY-ONSET DIABETES OF THE YOUNG (MODY) AND YOUNG ONSET TYPE 2 DIABETES FAILS TO DETECT ROBUST URINARY BIOMARKERS**

Anna L Gloyn^1,2^*, Johan H Faber^3*^, Daniel Malmodin^3^, Gaya Thanabalasingham^1,2^, Francis Lam^4^, Per Magne Ueland^5^, Mark I McCarthy^1,2,6^, Katharine R Owen^1,2**^, Dorrit Baunsgaard^3**^

**Supplementary Online Text S1**

**Materials and Methods**

**Sample preparation for UPLC-MS and NMR**

Prior to UPLC-MS and NMR analysis the samples were thawed at 4°C before centrifugation at 10,000*g* for 15 min at 4°C. The urine samples were prepared in successive well plates including control urine samples and blank water. For NMR 60 μl urine was diluted with 30 μl 2 in 1 in phosphate buffer (0.3 M, 20% D_2_O, pH 7.4) supplemented with 1 mM trimethylsilyl-2,2,3,3-tetradeuteropropionic acid (TSP) as internal chemical shift standard and 0.1% (w/v) sodium azide to avoid bacterial contamination. Samples were analyzed in triplicate for both analysis methods.

**UPLC-MS**

The UPLC Q-Tof MS comprised an AQUITY UPLC setup equipped with a binary solvent delivery manager, a sample manager, and a sample organizer hyphenated to a Q-TOF Premier mass spectrometer (Waters, Milford, USA). The system was operated using MassLynx software (version 4.0, Waters, Milford, USA). Liquid chromatography was performed with a UPLC-BEH C18 column, of dimensions 2.1 × 100 mm (Waters, Milford, USA). The column was maintained at 40°C. Gradient elution was employed with a mobile phase of A = 0.1% formic acid and B = acetonitrile + 0.1% formic acid. Urinary metabolites were eluted from the UPLC column using the following gradient: 0 to 0.4 min: 2% B; 0.4 to 2 min: 2% to 50% B; 2 to 9 min: 50 to 100% B. The flow rate was 0.6 ml/min. The samples were maintained at 4°C and 5 μL of each sample was injected onto the column.

Electrospray ionization mass spectrometry was performed in positive ion mode. The Q-Tof mass spectrometer was operated with a capillary voltage of 3.2 kV, cone voltage of 35, a MCP detector voltage of 1,990 V, a desolvation gas flow of 850 l/h, a cone gas flow of 20 l/h, a desolvation temperature of 350°C, and a source temperature of 120°C. Mass spectra were recorded in the range 50 to 850 *m/z* with an acquisition rate set at 0.08 s and a 0.02 s interscan delay. Leucine-enkephalin was employed as the lock mass ([M+H]^+^ ion at *m/z* 556.2771) at a concentration of 20 ng/ml (in 0.1% formic acid) at a flow rate of 20 μl/min via a lockspray interface. For sequential MS/MS experiments, argon was employed as the collision gas and collision energy was set to 32 eV. MS Data was collected in centroid mode.

**^1^H NMR spectroscopy**

^1^H NMR experiments were acquired on a Bruker DRX 600 spectrometer (Bruker BioSpin, Rheinstetten, Germany) operating at 600.13 MHz for 1H, equipped with a 30µl flow probe and a Gilson Liquid Handler (Gilson Inc., Middleton, WI). 1D spectra were acquired at 300 K using a standard 1-dimensional ^1^H experiment [RD – Gz– 90° – 3 μs – 90° – tm – Gz– 90° – acquire], incorporating the first increment of a 2D NOESY pulse sequence. Water suppression was achieved with continuous wave irradiation on the water peak during the 2.04 s recycle delay (RD) and 100 ms mixing time (tm). 128 free induction decays (FIDs) were accumulated collecting 64k data points with a spectral width of 20 ppm. The FIDs were zerofilled to 128k and apodized by an exponential window function using a line broadening factor of 0.3 Hz before Fourier transformation.

**Peak picking using XCMS software**

The R package XCM (v. 2.8, [www.r-project.org](http://www.r-project.org/)) was used to pick peaks in the data files and to match the picked peaks across samples. Prior to peak picking, the sample files were assigned to the classes, HNF1A, GCK, T2D, normoglycaemic controls, control urine and blank water. Optimization of the input parameters for the filtration and peak identification command xcmsSet was done from visual inspection of the raw data files in MassLynx, and of the peaks as they were picked in XCMS. The command was run with the profile setting set to ‘bin’. Only peaks eluted after 20 seconds to 6.5 minutes of retention time were considered for further analysis. The input bandwidth parameter for the group command matching the peaks across the samples was decided by running the command and then plotting the resulting groups. A relatively high number of peaks in at least one class were required to form a group in the first grouping. Optimization of the input parameters for the retention time correction command ‘retcor’ was done by running the function and plotting the result. Relatively conservative corrections were sought, since it was observed from the various groupings using the grouping command that the peaks eluted at approximately the same time in the different spectra. A relatively large number of peaks were allowed to be missing in the peak groups used for alignment due to the large number of water, pure water and test mix samples lacking most peaks. The final retention time corrections correlated well with the order the samples had been run and their sizes about a couple of seconds at the most. A second grouping of the corrected peaks was subsequently performed with relaxed requirements regarding how many peaks had to be found in a particular class of samples. Peak intensities were calculated for missing peak data using the ‘FillPeaks’ command.
