# Supplementary material for: Metabolic Profiling in Maturity-Onset Diabetes of the Young (MODY) and Young Onset Type 2 Diabetes Fails to Detect Robust Urinary Biomarkers
Source: PLoS One. 2012 Jul 30;7(7):e40962. doi: 10.1371/journal.pone.0040962 (PMC3408469; doi:10.1371/journal.pone.0040962)
Supplement: Table S1 — Q2Y (goodness of fit) for PLS-DA models. (DOC) [file pone.0040962.s009.doc]

**METABOLIC PROFILING IN MATURITY-ONSET DIABETES OF THE YOUNG (MODY) AND YOUNG ONSET TYPE 2 DIABETES FAILS TO DETECT ROBUST URINARY BIOMARKERS**

**Supplementary Online Information**

**Table S1. Q^2^Y (goodness of fit) for PLS-DA models**

| **Model** | **Q^2^Y** | **Components** | **Permutation test validity** |
| --- | --- | --- | --- |
| 4-class model (Controls, HNF1A, GCK and T2D) | 0.19 | 1 | No |
| 2-class model (Controls = 1 class; HNF1A, GCK and T2D = 1 class) | 0.55 | 2 | Yes |
| 3-class model (HNF1A, GCK and T2D) | 0.011 | 2 | No |
| 2-class model (HNF1A = 1 class; GCK and T2D = 1 class) | 0.52 | 3 | Yes |
