# Supplementary material for: Metabolic Profiling in Maturity-Onset Diabetes of the Young (MODY) and Young Onset Type 2 Diabetes Fails to Detect Robust Urinary Biomarkers
Source: PLoS One. 2012 Jul 30;7(7):e40962. doi: 10.1371/journal.pone.0040962 (PMC3408469; doi:10.1371/journal.pone.0040962)
Supplement: Table S2 — Amino acid concentrations in urine measured by 1H NMR in the diabetic subgroups. (DOC) [file pone.0040962.s010.doc]

**METABOLIC PROFILING IN MATURITY-ONSET DIABETES OF THE YOUNG (MODY) AND YOUNG ONSET TYPE 2 DIABETES FAILS TO DETECT ROBUST URINARY BIOMARKERS**

**Supplementary Online Information**

**Table S2. Amino acid concentrations in urine measured by ^1^H NMR in the diabetic subgroups**.

|  | **HNF1A** | **GCK** | **T2D** | **p** | **p** |
| --- | --- | --- | --- | --- | --- |
|  |  |  |  | **(HNF1A vs. GCK)** | **(HNF1A vs. T2D)** |
| **Alanine (μM)** | 217±147 (13) | 131±81 (17) | 131±121 (14) | 0.07 | 0.11 |
| **Glycine (μM)** | 718±572 (13) | 487±243 (17) | 317±233 (14) | 0.19 | 0.03 |
| **Histidine(μM)** | 614±384 (6) | 377±228 (8) | 474±217 (6) | 0.20 | 0.50 |
| **Lysine (μM)** | 133±138 (12) | 90±100 (17) | 163±238 (12) | 0.34 | 0.71 |
| **Methionine (μM)** | 83±99 (13) | 40±26 (16) | 55±48 (14) | 0.15 | 0.37 |
| **Phenylalanine (μM)** | 382±213 (13) | 438±312 (17) | 402±285 (14) | 0.56 | 0.75 |
| **Threonine (μM)** | 97±54 (13) | 163±67 (17) | 121±141 (14) | <0.01 | 0.34 |
| **Tryptophan (μM)** | 49±31 (13) | 42±30 (17) | 34±22 (14) | 0.56 | 0.16 |
| **Tyrosine (μM)** | 66±34 (13) | 61±39 (17) | 56±34 (14) | 0.74 | 0.46 |
| **Valine (μM)** | 36±20 (13) | 23±11 (17) | 21±13 (14) | 0.04 | 0.02 |

Data are relative NMR concentrations given as mean ± SD; (n) = number of subjects; p-value is calculated using two-tail unpaired t-test (p < 0.05 is considered significant). NMR peaks of amino acids have been integrated relatively to an internal standard. The number of subjects vary in the respective groups for each amino acid because of overlapping peaks in some of the spectra compared to others (could be overlapping peaks from glucose in some of the spectra).
